# Supplementary material for: Glycosaminoglycans as Biomarkers for Mucopolysaccharidoses and Other Disorders
Source: Diagnostics (Basel). 2021 Aug 28;11(9):1563. doi: 10.3390/diagnostics11091563 (PMC8468223; doi:10.3390/diagnostics11091563)
Supplement: Supplementary file 1 [file diagnostics-11-01563-s001.zip › diagnostics-1267898-supplementary.pdf]

Supplementary Materials:

Table S1: Patients with disease or condition and corresponding glycosaminoglycan levels (ng/mL).

Sex	Age	Diagnosis	Severity	Disease Stage	DiHS-0S	DiHS-NS	Di4S	Di-sulfated KS	Mono-sulfated KS	DiKS/ Total KS
M	0.33	rhabdomyolysis, UTI	mild	initial	21.5	6.4	110.5	22.2	421.1	5.0
M	0.33	rhabdomyolysis, UTI	mild	recovery	16.6	8.2	96.9	108.4	420.3	20.5
M	0.0	hypertrophic cardiomyopathy	moderate	screening (stable)	25.7	21.6	152.3	59.2	549.5	9.7
M	0	left megalocephaly	severe	initial	12.4	14.5	49.4	61.6	384.9	13.8
M	0.0	developmental disorder	unknown	unknown	36.5	18.2	104.0	147.7	912.1	13.9
unknown	0.04	GAII suspect	mild	screening (stable)	56.1	16.7	113.8	260.6	1095.1	19.2
M	0.04	jaundice, metabolic acidosis	mild	peak	42.4	14.8	151.8	89.2	550.7	13.9
M	0.07	ALTE, hypothermia, hypoglycemia	moderate	recovery	81.7	22.6	138.7	330.8	1254.2	20.9
M	0.07	hypoglycemic encephalopathy	severe	screening (stable)	26.9	17.9	122.0	281.2	1015.0	21.7
F	0.08	acute myocarditis	severe	initial	30.3	0.8	46.8	80.4	513.2	13.5
M	0.08	early myoclonic encephalopathy?	severe	peak	16.8	2.0	23.6	117.0	252.8	31.6
M	0.08	early myoclonic encephalopathy?	severe	peak	27.3	1.7	121.2	97.7	838.4	10.4
F	0.08	epilepsy, seizure	unknown	screening (stable)	25.2	25.3	101.8	162.8	955.7	14.6
F	0.17	acute encephalopathy	severe	initial	65.1	131.0	542.6	232.7	699.4	25.0
F	0.2	encephalopathy, RSV +, hepatitis	severe	initial	38.1	37.5	236.1	458.6	1458.3	23.9
F	0.17	secondary carnitine deficiency	mild	screening (stable)	31.3	19.7	114.5	326.1	1002.1	24.6
F	0.25	afebrile seizure, epilepsy?	moderate	screening (stable)	30.9	24.0	120.3	153.7	918.1	14.3
F	0.25	epilepsy	moderate	peak	4.6	7.1	45.3	129.1	637.3	16.8
F	0.25	coarctation of the aorta, lactic acidemia, CK elevation	moderate	screening (stable)	25.2	10.8	68.2	92.5	584.6	13.7
F	0.3	influenza encephalopathy	unknown	unknown	112.9	353.1	928.5	630.1	1193.0	34.6
F	0.3	influenza encephalopathy	unknown	unknown	68.5	158.0	444.9	483.6	945.8	33.8

F	0.25	encephalopathy	severe	initial	44.5	62.2	296.6	240.9	818.9	22.7
M	0.25	acute encephalopathy, DIC	moderate	initial	60.8	210.1	495.2	336.8	554.3	37.8
M	0.33	acute encephalopathy	severe	initial	28.3	53.8	237.6	330.2	967.2	25.5
M	0.33	encephalopathy?, lactic acidemia	severe	unknown	19.0	17.4	96.6	94.4	524.2	15.3
F	0.3	CPT2 deficiency	moderate	peak	31.3	20.5	163.3	194.1	1109.8	14.9
F	0.33	West syndrome	moderate	screening (stable)	24.2	20.1	66.1	78.8	570.5	12.1
F	0.33	shock, encephalopathy, Reye syndrome?, CPT2 deficiency	severe	peak	30.0	59.8	267.0	187.9	519.4	26.6
F	0.42	hyperbilirubinemia, transient tachypnea	moderate	screening (stable)	32.3	16.2	130.8	140.9	674.3	17.3
F	0.42	West syndrome	moderate	screening (stable)	35.2	16.2	137.6	312.8	1165.2	21.2
unknown	0.42	seizure	moderate	screening (stable)	13.4	18.2	121.7	117.5	762.0	13.4
F	0.42	partial convulsion	moderate	screening (stable)	16.5	51.1	56.4	98.6	488.2	16.8
F	0.42	muscle weakness, low-oxygen ischemic injury?	mild	screening (stable)	15.4	24.8	127.2	81.1	662.9	10.9
M	0.42	SIDS?, metabolic acidosis	moderate	initial	20.7	37.6	42.4	126.0	725.2	14.8
M	0.4	GAII suspect	unknown	screening (stable)	26.5	12.4	96.6	314.1	741.9	29.7
unknown	0.42	GAII	unknown	screening (stable)	21.7	12.4	114.4	85.8	789.3	9.8
M	0.42	metabolic acidosis	mild	screening (stable)	26.5	5.7	44.0	87.3	544.0	13.8
M	0.42	hypoxic ischemic encephalopathy	severe	peak	1.6	0.8	57.8	15.1	266.4	5.3
M	0.42	acute encephalopathy	severe	initial	30.6	33.4	188.7	216.3	769.4	21.9
F	0.42	acute encephalopathy with biphasic seizures and late reduced diffusion (suspect)	moderate	initial	30.3	19.8	148.8	338.8	909.6	27.1
M	0.42	acute encephalopathy suspect	moderate	initial	20.7	9.8	134.6	148.7	687.4	17.8
M	0.5	vomiting	mild	screening (stable)	23.4	11.8	81.8	199.3	550.5	26.6
M	0.5	TAM, elevation of AST, ALT	mild	screening (stable)	16.7	13.7	100.4	77.9	488.4	13.8
M	0.5	epilepsy, seizure	mild	screening (stable)	19.3	24.1	137.0	171.8	846.1	16.9

F	0.5	acute encephalopathy	severe	peak	61.2	393.1	1128.9	235.7	592.0	28.5
M	0.5	hemorrhagic shock and encephalopathy syndrome	severe	initial	9.5	20.9	117.3	107.6	454.8	19.1
F	0.58	developmental delay, renal failure	mild	screening (stable)	19.7	7.9	62.6	172.6	476.4	26.6
M	0.58	acute encephalopathy	moderate	screening (stable)	17.5	11.0	99.8	242.4	750.9	24.4
F	0.58	acute encephalopathy	moderate	recovery	30.2	13.9	131.0	152.7	603.9	20.2
F	0.58	acute encephalopathy	severe	peak	58.9	97.0	418.3	239.3	874.6	21.5
F	0.58	floppy infant	mild	screening (stable)	11.6	17.2	88.5	110.7	675.8	14.1
F	0.67	muscle weakness	mild	screening (stable)	24.7	16.3	101.2	98.3	782.6	11.2
M	0.67	hyper-CK-emia, pneumonia, developmental delay, hypotonic	mild	screening (stable)	11.6	12.5	80.7	108.7	461.2	19.1
M	0.67	Acute encephalopathy	severe	initial	70.5	253.2	782.8	175.1	557.9	23.9
M	0.7	post ALTE, cardiac arrest, brain edema, on hypothermia therapy	severe	peak	27.2	25.2	107.4	82.0	553.1	12.9
F	0.75	rotavirus gastroenteritis, generalized seizure	unknown	unknown	27.8	20.7	123.9	192.9	841.1	18.7
M	0.8	AGE, dehydration, liver dysfunction	moderate	peak	19.4	7.3	71.6	144.7	508.8	22.1
M	0.75	jaundice	mild	screening (stable)	19.4	10.8	73.6	259.7	941.5	21.6
F	0.75	development disorders, hypoglycemia	mild	screening (stable)	27.4	13.1	82.4	144.8	452.8	24.2
M	0.8	MR	mild	screening (stable)	21.5	10.7	79.4	111.1	607.8	15.5
F	0.75	acute encephalopathy	moderate	initial	42.5	46.8	243.5	344.2	867.8	28.4
F	0.75	Leigh encephalopathy suspect	moderate	peak	12.5	10.1	50.7	94.7	483.6	16.4
M	0.75	mitochondrial disease suspect, Leigh encephalopathy	severe	peak	23.6	19.2	123.6	111.1	572.7	16.2

F	0.8	norovirus encephalopathy, artificial respiration +	severe	peak	61.4	54.5	249.3	305.3	630.8	32.6
F	0.83	developmental disorder	mild	screening (stable)	35.7	44.9	49.5	93.9	607.1	13.4
F	0.83	developmental disorder	mild	screening (stable)	21.0	11.8	75.5	124.3	942.1	11.7
M	0.83	methylmalonic acidemia	moderate	screening (stable)	22.9	11.9	86.0	63.1	396.7	13.7
M	0.83	High CKemia	unknown	screening (stable)	17.0	8.1	62.0	138.2	769.6	15.2
M	0.8	fatty acid metabolism disorder suspect	unknown	screening (stable)	18.5	9.0	63.8	118.5	931.4	11.3
M	0.92	epilepsy	mild	screening (stable)	12.1	8.4	65.3	88.4	551.6	13.8
M	0.92	developmental delay, hypopituitary gland	mild	screening (stable)	6.8	6.5	49.3	73.4	324.0	18.5
M	0.92	West syndrome	moderate	screening (stable)	46.7	20.1	173.2	363.5	818.0	30.8
M	1	myopathy, rhabdomyolysis?	mild	screening (stable)	21.6	9.4	72.7	133.3	892.4	13.0
F	1.0	secondary carnitine deficiency, CFPN-PI induced	moderate	peak	23.3	16.6	102.2	189.7	1190.9	13.7
F	1.0	MCAD	moderate	initial	6.0	9.7	58.1	54.7	319.1	14.6
M	1.0	fatty acid metabolism disorder suspect	mild	screening (stable)	22.9	4.8	72.1	132.8	786.2	14.4
M	1.0	CPT2 deficiency	moderate	screening (stable)	12.2	7.4	57.4	198.6	515.9	27.8
M	1	secondary carnitine deficiency, mental deficiency, epilepsy	mild	screening (stable)	20.6	14.0	79.7	134.0	878.5	13.2
F	1.00	inguinal hernia, liver disorder	mild	screening (stable)	22.7	4.7	23.6	102.8	634.5	13.9
F	1	hypoglycemia, congenital biliary atresia	moderate	recovery	23.3	21.6	66.0	100.9	617.5	14.0
M	1	a bilious attack, disturbance of consciousness, fatty liver	moderate	recovery	9.1	24.2	89.9	183.1	1229.5	13.0
F	1	afebrile seizure, epilepsy?	mild	screening (stable)	14.1	2.9	75.4	138.4	871.1	13.7

F	1	afebrile convulsion, hypoglycemia	moderate	screening (stable)	27.9	22.6	93.9	94.0	605.9	13.4
M	1.0	tonic-clonic seizure	mild	screening (stable)	16.3	8.8	68.0	100.7	576.9	14.9
M	1	ketotic hypoglycemia	moderate	recovery	33.4	11.2	99.9	139.0	840.2	14.2
M	1.00	ketotic hypoglycemia	moderate	initial	16.3	9.7	84.5	199.7	558.9	26.3
M	1.00	low glucose	mild	screening (stable)	20.5	6.1	28.4	68.7	407.7	14.4
F	1	hyper-CK-emia, muscular dystrophy?	mild	screening (stable)	13.1	9.9	60.6	157.7	717.1	18.0
unknown	1	metabolic myopathy suspect	mild	screening (stable)	22.8	11.3	84.3	83.8	632.0	11.7
F	1.0	mental and development disorder	mild	screening (stable)	22.5	12.7	61.2	125.5	830.3	13.1
F	1.0	developmental disorder, mitochondria disorder?	mild	screening (stable)	19.8	13.3	80.2	120.5	798.1	13.1
M	1.0	developmental delay	mild	screening (stable)	25.3	30.0	188.7	121.8	1071.7	10.2
M	1.0	developmental disorder, hyperlactatemia	mild	screening (stable)	23.0	10.6	62.7	165.7	1095.1	13.1
M	1.00	developmental disorders	mild	screening (stable)	8.9	4.6	31.4	84.9	967.9	8.1
F	1	development disorder, febrile delirium, metabolic disorders?	mild	screening (stable)	24.0	1.0	84.5	129.8	723.3	15.2
F	1	hyper-CK-emia	mild	screening (stable)	21.3	1.6	43.9	112.4	597.0	15.8
M	1	rotavirus encephalopathy, encephalitis, myocarditis	severe	screening (stable)	5.4	5.3	58.9	208.7	749.8	21.8
M	1.0	MR, microcephaly, hyperlactatemia	mild	screening (stable)	20.4	9.2	66.6	115.4	828.3	12.2
unknown	1.0	head circumference enlargement, MR	mild	screening (stable)	22.0	3.3	64.2	334.4	988.8	25.3
M	1.00	metabolic acidosis	moderate	initial	15.1	5.6	40.8	48.7	334.2	12.7
M	1	SIDS-like, influ A+, acidosis, hyperlactatemia	severe	initial	46.2	2828.8	12475.3	285.1	768.0	27.1
M	1	developmental delay, rota virus	mild	screening (stable)	20.8	12.2	82.6	139.0	857.6	13.9
F	1.0	HHV-6 encephalopathy	severe	peak	48.8	64.0	309.2	260.3	854.8	23.3

F	1.0	Virus acute encephalopathy	severe	peak	84.6	109.6	403.1	433.7	1150.2	27.4
F	1.0	Exanthema subitem encephalopathy	severe	initial	42.2	67.5	345.3	353.5	1061.3	25.0
M	1.0	HHV6 encephalopathy suspect	moderate	initial	32.8	16.7	148.2	166.0	714.2	18.9
M	1	acute encephalopathy with biphasic seizures and late reduced diffusion, HHV6	moderate	unknown	17.6	3.9	90.9	121.1	724.8	14.3
F	1	influenza encephalopathy (A+ suspect)	moderate	peak	28.5	10.0	56.1	312.1	825.2	27.4
M	1	acute encephalopathy	moderate	recovery	30.6	49.6	134.3	191.6	663.1	22.4
M	1	hypoxic ischemic encephalopathy	moderte	recovery	24.0	4.4	57.1	212.5	389.7	35.3
M	1	acute encephalopathy	moderate	initial	30.8	1.2	47.0	180.2	451.9	28.5
F	1	acute encephalopathy	moderate	screening (stable)	45.9	12.0	116.1	321.4	1031.7	23.8
M	1.0	acute encephalopathy with biphasic seizures and late reduced diffusion	severe	initial	20.4	2.4	83.4	54.6	489.4	10.0
F	1	acute encephalopathy suspect	unknown	initial	25.2	11.9	84.1	488.2	1124.3	30.3
F	2	periventricular leukomalacia, cerebral palsy, rhabdomyolysis	mild	initial	21.0	27.8	157.6	225.9	861.9	20.8
M	2	artificial respiration, plumonary hypertension crisis	severe	peak	11.1	13	96	149.8	424.3	26.1
M	2	asthma, lactacidemia	mild	screening (stable)	17.5	7.9	36.1	134.6	719.1	15.8
unknown	2.00	asthma, lactacidemia	mild	screening (stable)	16.2	6.4	39.9	184.2	1198.7	13.3
F	2.0	VLCAD suspected, repeated hypoglycemia	moderate	recovery	8.3	8.4	48.5	162.7	929.7	14.9
F	2.0	carnitine deficiency suspect	mild	screening (stable)	5.8	5.9	60.9	128.0	746.9	14.6

F	2	shock, congenital heart disease (PA, VSD post op.), carnitine deficiency, hypoglycemia	moderate	peak	95.1	303.5	1022.1	263.2	850.3	23.6
F	2	ketotic hypoglycemia, vomiting	mild	initial	17.6	0.5	21.2	113.1	612.3	15.6
M	2	liver dysfunction	moderate	recovery	35.5	12.3	89.2	81.8	821.5	9.1
F	2	severe myoclonic epilepsy	severe	recovery	21.8	13.9	61.8	85.3	393.6	17.8
M	2	left-handed convulsion	mild	screening (stable)	11.2	10.2	72.0	178.1	1044.2	14.6
F	2	convulsion, hypoglycemia	moderate	recovery	28.0	9.0	65.8	380.7	1060.2	26.4
M	2	convulsive seizure	severe	peak	34.3	16.6	130.6	174.9	1156.4	13.1
M	2	febrile convulsion	mild	recovery	24.0	15.4	58.3	201.5	917.2	18.0
F	2	convulsive seizure	severe	recovery	33.6	18.8	110.8	229.8	736.3	23.8
M	2	hypoglycemic attack	moderate	screening (stable)	12.3	7.6	36.8	195.3	1229.8	13.7
F	2	MR	mild	screening (stable)	17.8	8.1	77.8	143.8	670.4	17.7
F	2	HHV-6 encephalopathy	mild	screening (stable)	25.4	5.3	42.9	73.7	659.1	10.1
F	2	viral encephalopathy, influenza	moderate	recovery	12.9	7.4	40.1	87.5	375.4	18.9
M	2.0	encephalopathy, unknown virus-related	severe	peak	15.6	18.9	101.4	174.3	1201.3	12.7
M	2	acute encephalopathy	moderate	peak	15.1	14.7	92.6	257.3	917.4	21.9
M	2	acute encephalopathy	severe	recovery	36.1	0.7	71.9	172.0	509.1	25.3
M	2	acute encephalopathy	moderate	screening (stable)	26.2	18.8	98.4	120.0	768.2	13.5
F	2	Leigh encephalopathy	mild	peak	18.6	1.4	64.5	124.7	448.7	21.7
F	2	epilepsy, acute encephalopathy with biphasic seizures and late reduced diffusion	severe	peak	3.4	9.3	79.8	130.4	579.6	18.4
M	2.0	secondary carnitine deficiency, post hypoglycemia (glu 16 mg/dl)	moderate	recovery	20.4	10.5	68.3	448.9	1263.7	26.2
F	3	rhabdomyolysis, Hypernatremia, Adeno, Rota +	moderate	recovery	18.3	12.3	63.0	123.8	817.8	13.1

M	3.0	carnitine deficiency	mild	recovery	32.9	19.8	115.0	88.5	419.8	17.4
F	3.0	hypocarnitinemia, under carnitine administration	mild	screening (stable)	24.6	9.0	69.2	134.4	901.6	13.0
M	3.0	cyclic vomiting	moderate	recovery	16.9	7.7	54.9	133.3	896.9	12.9
F	3.00	afebrile convulsion	mild	screening (stable)	13.5	19.2	24.5	126.2	604.0	17.3
F	3	hypoglycemia attack	mild	screening (stable)	18.1	4.0	58.9	121.4	787.9	13.4
F	3	Low glucose	mild	screening (stable)	30.7	5.8	99.0	220.9	1149.3	16.1
F	3.0	ketotic hypoglycemia, impaired consciousness	moderate	screening (stable)	32.9	8.6	29.9	118.6	612.3	16.2
M	3	rota virus encephalopathy	moderate	recovery	13.0	12.5	57.9	118.0	660.4	15.2
M	3	rota virus encephalopathy	moderate	recovery	0.8	0.5	3.1	75.5	659.4	10.3
M	3.0	encephalopathy (virus unknown)	severe	recovery	1.5	5.6	58.8	104.0	626.5	14.2
M	3	acute encephalopathy with biphasic seizures and late reduced diffusion, unknown virus	severe	recovery	7.8	5.4	64.9	104.6	515.8	16.9
M	3.0	rotavirus encephalopathy	severe	recovery	1.1	4.7	64.5	175.3	364.2	32.5
M	3	influenza encephalopathy (B+)	moderate	initial	17.9	17.7	88.3	377.4	757.2	33.3
M	3	influenza encephalopathy (A+)	severe	peak	68.9	638.6	1212.0	323.2	293.5	52.4
unknown	3	influenza encephalopathy	severe	initial	158.5	1933.9	3226.7	516.1	811.6	38.9
F	3.0	acute encepalopathy, brain edema, VLCAD deficiency, influenza	moderate	recovery	13.5	8.9	31.0	179.2	880.1	16.9
F	3.0	rota virus encephalopathy	severe	peak	29.5	16.3	93.3	237.5	754.1	23.9
F	3	acute encephalopathy with biphasic seizures and late reduced diffusion	moderate	screening (stable)	15.4	8.4	64.1	285.0	853.1	25.0
M	3	epileptic encephalopathy	moderate	screening (stable)	29.2	5.5	63.8	199.9	764.7	20.7

F	3	acute encephalopathy	moderate	initial	25.0	25.0	156.3	288.0	689.4	29.5
M	3	hypoxic ischemic encephalopathy	moderate	initial	39.0	23.8	109.6	354.8	575.7	38.1
M	4.00	rhabdomyolysis	mild	peak	23.9	10.8	92.0	210.6	1032.6	16.9
M	4.00	bronchitis, hypoglycemia	moderate	screening (stable)	18.7	6.2	28.0	149.4	775.3	16.2
F	4.0	unknown virus related hepatitis (herpangina)	moderate	recovery	15.9	14.1	67.7	188.1	1042.3	15.3
F	4.0	reccurent vomitting, hypoglycemia, developmental delay	mild	initial	20.4	8.3	60.0	409.4	1126.7	26.6
M	4	liver disorder	moderate	screening (stable)	19.6	16.9	101.2	119.2	697.1	14.6
F	4.0	hypoglycemia	mild	screening (stable)	27.9	10.9	73.5	160.7	1054.5	13.2
unknown	4	Hypre-CK-emia	mild	screening (stable)	40.2	7.2	52.2	138.6	611.1	18.5
M	4	GA II suspect	unknown	screening (stable)	33.0	7.3	89.4	706.4	1384.0	33.8
M	4	hyperlactatemia	moderate	recovery	20.2	7.2	62.9	274.3	660.0	29.4
M	4.0	epilepsy, influenza encephalopathy, metabolic disorder?	moderate	screening (stable)	20.1	0.7	28.1	120.9	546.9	18.1
F	4	hypoxic ischemic encephalopathy	moderate	recovery	25.1	27.7	188.5	278.5	622.4	30.9
F	4	acute encephalopathy	moderate	initial	40.9	5.1	64.7	148.8	582.7	20.3
F	4	acute encephalopathy	moderate	initial	20.6	8.9	57.9	99.3	399.5	19.9
M	5.0	secondary carnitine deficiency	mild	recovery	14.9	6.6	50.2	456.7	1067.8	30.0
F	5.0	unconsciousness, vomiting	moderate	recovery	13.1	3.2	48.0	111.0	764.2	12.7
F	5.00	hepatosplenomegaly	moderate	screening (stable)	24.3	6.4	33.8	61.9	324.6	16.0
F	5	ketoacidosis, hypoglycemia	moderate	initial	2.7	9.6	55.6	111.7	676.8	14.2
F	5.0	hyperlactatemia	mild	screening (stable)	29.3	24.2	82.3	194.9	923.8	17.4
M	5	periodic paralysis suspect, GAII suspect	mild	screening (stable)	19.1	11.3	85.7	176.8	1025.4	14.7

F	5	acute encephalopathy with biphasic seizures and late reduced diffusion (B+)	moderate	initial	29.4	22.1	123.5	638.9	1139.9	35.9
M	5	acute encephalopathy	moderate	initial	29.3	15.7	84.6	253.0	686.4	26.9
M	5.0	hypoxic ischemic encephalopathy	severe	unknown	16.9	8.5	63.1	241.9	586.0	29.2
M	5.0	Leigh encephalopathy (suspect)	mild	initial	27.5	16.5	94.3	184.9	635.4	22.5
M	5	Ifosfamide encephalopathy	unknown	unknown	23.5	13.5	101.4	500.9	656.8	43.3
M	5	febrile convulsion, ADHD, encephalopathy, hypotonic	mild	peak	19.9	11.3	64.5	105.4	659.6	13.8
F	6.0	chronic lung disease, cyclic vomiting	mild	unknown	22.3	5.1	27.0	213.3	907.8	19.0
M	6	cyclic vomiting	mild	initial	12.7	11.8	50.4	171.9	757.7	18.5
F	6.0	lissencephaly with Infantile spasms	moderate	screening (stable)	24.8	6.9	71.3	226.6	639.1	26.2
F	6	complex partial status epilepsy	moderate	screening (stable)	16.8	42.6	27.3	84.0	457.8	15.5
F	6	viral acute encephalopathy, hypophosphatasia	moderate	recovery	24.1	18.4	108.7	330.2	643.4	33.9
F	6	influenza encephalopathy suspect	unknown	unknown	18.5	5.5	69.9	140.7	719.1	16.4
F	6.0	influenza encephalopathy (B+ suspect)	moderate	initial	19.8	9.8	62.7	394.2	705.4	35.8
M	6.0	influenza encephalopathy	moderate	recovery	17.3	32.6	30.0	87.1	404.7	17.7
M	6	acute focal bacterial nephritis encephalopathy suspect	moderate	screening (stable)	33.5	24.2	139.8	430.7	1134.9	27.5
M	7	myositis due to influenza, rhabdomyolysis	moderate	peak	19.9	8.7	76.2	166.8	833.7	16.7
M	7.0	VLCAD	mild	peak	19.6	12.4	65.2	233.1	973.1	19.3
M	7.0	cyclic vomiting	mild	screening (stable)	16.2	6.4	52.0	116.1	711.8	14.0

M	7	recurrent liver dysfunction	mild	screening (stable)	19.6	9.3	64.3	390.3	966.6	28.8
F	7	mental deficiency, neurogenic paralysis, epilepsy	moderate	screening (stable)	14.3	3.9	41.7	157.0	666.0	19.1
M	7	partial seizure, West syndrome, neonatal asphyxia	moderate	screening (stable)	14.4	12.0	48.3	143.0	649.1	18.1
unknown	7	FDPase deficiency suspect, ketotic hypoglycemia	unknown	screening (stable)	17.8	8.4	80.7	446.6	925.8	32.5
M	7.0	hyper-CK-emia	mild	initial	27.9	37.5	24.5	113.0	506.8	18.2
M	7	acute encephalopathy	severe	recovery	12.0	16.7	88.9	115.9	456.1	20.3
M	7	encephalopathy	moderate	screening (stable)	2.2	1.5	31.6	73.1	527.8	12.2
F	7	acute encephalopathy	unknown	unknown	11.6	9.0	55.4	400.7	769.7	34.2
M	7	acute encephalopathy, seizure	moderate	recovery	11.1	4.7	71.5	139.6	591.5	19.1
M	8.0	kidney stone	unknown	screening (stable)	24.3	6.4	54.3	153.1	857.1	15.2
M	8.00	vomiting, influenza	mild	initial	15.3	7.9	19.7	121.4	669.4	15.4
M	8.0	acetonemic vomiting	mild	recovery	22.0	7.8	52.3	162.7	905.0	15.2
M	8	cyclic vomiting, a bilious attack	mild	screening (stable)	21.7	7.0	60.1	175.6	1008.7	14.8
M	8.0	Asperger syndrome, epileptic seizure, EEG, Roland epilepsy suspect	mild	screening (stable)	4.6	9.4	56.7	152.4	676.3	18.4
F	8.00	hyperammonemia, febrile convulsion, influenza A	moderate	peak	11.1	74.3	292.6	840.7	25.8	97.0
M	8.0	influenza encephalopathy	moderate	screening (stable)	16.5	7.1	68.6	159.5	752.9	17.5
F	8	influenza encephalopathy	moderate	recovery	29.9	5.7	65.8	148.1	422.8	25.9
M	8.0	influenza A+ encephalopathy, influenza pneumonia	moderate	recovery	29.9	2.9	97.3	271.9	607.4	30.9
M	8.0	influenza encephalopathy	moderate	recovery	22.2	15.0	40.3	139.2	627.9	18.1
F	8	encephalopathy, unknown virus	severe	recovery	33.1	5.6	59.5	160.2	696.5	18.7
M	8	acute encephalopathy	moderate	unknown	49.5	21.3	129.5	147.1	283.6	34.1

M	8	acute encephalopathy	severe	peak	29.2	15.1	126.9	357.2	859.7	29.4
M	8	acute encephalopathy	moderate	unknown	16.3	16.1	62.8	162.5	590.0	21.6
F	9.0	epilepsy, asthma	mild	screening (stable)	16.9	10.3	75.5	581.0	1043.9	35.8
M	9	acetonic vomiting	mild	initial	14.0	8.5	66.0	110.6	670.9	14.2
M	9	fulminant hepatic failure	severe	peak	31.3	23.7	130.2	154.9	491.6	24.0
F	9.0	MR	mild	screening (stable)	15.8	6.7	49.0	175.2	712.7	19.7
F	9	acute encephalopathy	moderate	recovery	22.1	13.1	68.2	161.4	452.2	26.3
F	9.5	influenza A+ encephalopathy	severe	peak	21.0	7.9	55.6	214.6	484.7	30.7
M	10	cyclic vomiting	mild	screening (stable)	22.9	9.0	76.6	405.6	998.8	28.9
unknown	10.0	recurrent myopathy	moderate	recovery	25.0	7.0	68.5	415.3	1133.9	26.8
F	10.0	hypoxic ischemic encephalopathy	moderate	initial	20.6	12.1	101.4	426.8	1005.7	29.8
M	10	acute encephalopathy, gall stone, choledocholithiasis	moderate	initial	19.2	19.7	158.5	243.5	663.6	26.8
M	11	vomiting, liver dysfunction	moderate	recovery	16.6	8.2	67.5	116.0	840.2	12.1
F	11.0	tonic-clonic seizure, rash	moderate	recovery	24.8	5.3	79.8	95.4	470.1	16.9
F	11	myoclonic epilepsy	mild	screening (stable)	12.6	6.5	42.2	145.1	807.6	15.2
F	11.0	myopathy, rhabdomyolysis	moderate	recovery	1.4	7.5	7.9	4.8	484.7	1.0
F	11	viral acute encephalopathy suspect	moderate	peak	27.6	21.2	195.3	194.2	359.6	35.1
M	11	influenza encephalopathy (A+)	moderate	recovery	20.8	10.9	108.7	731.3	1188.9	38.1
M	11	encephalopathy, metabolic disorder?, influenza B+	mild	recovery	21.6	10.2	73.8	335.1	722.6	31.7
F	12.0	mycoplasma pneumonia	mild	peak	2.2	16.9	83.9	101.5	504.1	16.8
M	12	influenza encephalopathy (B+)	severe	initial	18.5	136.9	826.7	152.4	217.8	41.2
M	12	influenza encephalopathy (A+)	mild	screening (stable)	85.0	13.5	84.4	235.8	832.1	22.1
M	12.0	influenza encephalopathy (A+)	moderate	screening (stable)	52.9	10.1	61.7	214.0	881.2	19.5
F	12	influenza encephalopathy (B+)	severe	initial	105.6	278.0	658.4	412.3	457.7	47.4

M	13	carnitine deficiency, renal tubular dysfunction, cardiomegaly	moderate	screening (stable)	20.1	5.7	44.5	124.8	687.7	15.4
M	13	rhabdomyolysis	moderate	screening (stable)	20.9	6.0	57.2	92.3	594.9	13.4
M	13.0	hematuria, hyper-CK-emia	mild	screening (stable)	19.1	9.7	51.7	190.7	806.5	19.1
F	13	influenza encephalopathy (B+)	moderate	initial	50.8	49.1	190.5	278.4	437.6	38.9
M	13	acute encephalopathy	mild	screening (stable)	15.4	0.4	12.7	154.4	613.8	20.1
M	14.0	carnitine deficiency suspect	mild	screening (stable)	21.2	5.0	42.4	100.9	589.0	14.6
M	14	fatty liver	mild	screening (stable)	18.9	9.2	68.7	146.7	655.7	18.3
M	14	metabolic myopathy suspect	mild	screening (stable)	18.2	6.4	41.1	78.4	525.3	13.0
F	14.0	CPA post influenza A	severe	peak	4.2	23.8	97.3	101.2	350.5	22.4
M	14.0	fever, influenza B+ encephalopathy, multiple organ failure, DIC	severe	peak	46.6	186.0	766.5	232.4	422.8	35.5
M	14	influenza encephalopathy (B+)	moderate	recovery	71.4	9.0	62.4	90.8	376.8	19.4
M	14	influenza encephalopathy (B+)	moderate	initial	78.0	13.4	84.3	115.1	388.2	22.9
F	14	acute encephalopathy	moderate	peak	33.2	7.9	75.0	96.4	296.4	24.5
M	14.2	influenza encephalopathy (A+)	moderate	initial	21.4	7.7	74.8	181.8	526.3	25.7
M	15.0	muscle weakness	mild	screening (stable)	1.1	7.1	35.9	81.0	307.5	20.9
F	15.0	ventricular tachycardia	moderate	screening (stable)	16.9	3.8	36.3	51.7	316.4	14.0
F	15	influenza A+ encephalopathy (suspect)	moderate	initial	20.8	24.5	84.4	255.6	329.2	43.7
M	15	acute lymphocytic leukemia, encephalopathy, hyperammonemia	moderate	recovery	15.5	14.7	77.9	74.5	296.7	20.1
M	17	hyper-CK-emia, severe muscle ache	moderate	peak	19.6	5.6	46.2	71.4	396.5	15.3
M	17.0	acute encephalopathy	mild	screening (stable)	24.8	6.7	34.6	70.5	148.6	32.2
M	20	acute encephalopathy	mild	screening (stable)	44.1	11.6	79.8	131.4	546.8	19.4

unknown	26	CP, epilepsy, MR, asthma, testicular cancer	mild	screening (stable)	15.6	5.6	48.6	32.6	204.6	13.7
F	26	hyper-CK-emia	moderate	screening (stable)	20.1	4.6	30.9	43.3	196.1	18.1
F	37	leukoencephalopathy suspect	mild	screening (stable)	17.2	4.7	32.5	105.5	185.5	36.2
F	40.0	encephalitis, hyper-CK-emia	mild	screening (stable)	14.5	6.2	39.7	33.4	226.5	12.8
F	41	Ifosfamide encephalopathy	unknown	unknown	43.6	7.5	45.5	64.9	204.5	24.1
M	43	leukoencephalopathy (CADASIL syndrome suspect)	mild	screening (stable)	3.8	9.8	43.5	136.4	369.7	26.9
M	54	hyper-CK-emia, stiffness of back	mild	screening (stable)	17.9	5.7	44.5	86.8	334.3	20.6
F	57	Ifosfamide encephalopathy	unknown	unknown	65.0	11.0	67.3	93.3	513.0	15.4
F	59.0	Ifosfamide encephalopathy	unknown	unknown	61.7	9.8	59.5	106.9	286.9	27.1
F	60	Ifosfamide encephalopathy	moderate	recovery	89.7	12.0	88.0	62.5	280.7	18.2
M	62	leukoencephalopathy	unknown	unknown	20.5	4.8	38.1	102.2	305.2	25.1

Abbreviations: M=male, F=female, DiHS-0S= *O*-sulfated heparan sulfate, DiHS-NS= *N*-sulfated heparan sulfate, Di4S= dermatan sulfate, KS= keratan sulfate

Table S2: Patients with mucopolysaccharidoses and corresponding glycosaminoglycan levels (ng/mL).

Age	Diagnosis	DiHS-OS	DiHS-NS	Di-4S	KS	Di KS	Mono KS	DiKS/Total KS
13.1	MPS I	47.8	9.8	1.9	1379.8	327.4	1379.8	19.2
13.09041096	MPS I	63.2	8.9	3.1	973.3	226.7	973.3	18.9
3.8	MPS II	140.5	84.7	170.6	1084.5	256.0	1084.5	19.1
3.8	MPS II	65.3	62.3	137.3	1878.8	403.1	1878.8	17.7
4	MPS II	219.0	72.3	222.8	1331.5	255.3	1331.5	16.1
4	MPS II	114.1	30.7	39.3	915.4	171.7	915.4	15.8
4	MPS II	185.4	55.0	180.1	1306.7	185.6	1306.7	12.4
4	MPS II	98.0	45.5	35.4	1085.4	182.2	1085.4	14.4
4.663	MPS II	222.1	18.1	6.2	1816.8	187.3	1816.8	9.3
9	MPS II	289.2	80.4	201.1	1640.7	525.5	1640.7	24.3
9	MPS II	110.2	34.5	78.2	1031.6	311.5	1031.6	23.2
5	MPS II	292.6	92.5	314.5	1143.9	249.8	1143.9	17.9
5	MPS II	80.5	25.2	45.7	1058.3	279.9	1058.3	20.9
7	MPS II	100.8	24.3	54.2	819.5	228.1	819.5	21.8
7	MPS II	176.2	50.4	198.3	1336.9	381.4	1336.9	22.2
8	MPS II	392.9	84.6	135.8	2198.3	525.2	2198.3	19.3
6	MPS II	147.9	43.7	80.9	2093.3	556.8	2093.3	21.0
7.7	MPS II	57.5	19.8	48.3	1556.9	458.0	1556.9	22.7
5.2	MPS II	80.0	18.6	43.7	1766.5	427.3	1766.5	19.5
9.9	MPS II	35.5	7.8	24.8	795.0	272.9	795.0	25.6
9.8	MPS II	198.5	45.5	287.8	1641.9	404.6	1641.9	19.8
9.5	MPS II	74.1	68.0	38.1	1775.0	426.0	1775.0	19.4
8.5	MPS II	111.2	71.6	89.7	1385.8	344.4	1385.8	19.9
9.3	MPS II	249.4	98.5	295.7	1619.5	402.3	1619.5	19.9
5	MPS II	197.4	53.9	143.1	1908.8	516.9	1908.8	21.3

6	MPS II	205.1	55.6	121.6	925.1	173.8	925.1	15.8
10.4	MPS II	249.2	80.1	397.5	1617.4	424.6	1617.4	20.8
10.7	MPS II	41.4	14.1	37.3	850.7	270.3	850.7	24.1
12.3	MPS II	71.7	18.1	39.9	1000.6	331.4	1000.6	24.9
10	MPS II	166.5	50.9	193.2	1279.6	266.0	1279.6	17.2
10	MPS II	69.7	19.0	26.6	613.2	139.0	613.2	18.5
11.1	MPS II	301.1	121.5	40.7	621.5	201.7	621.5	24.5
12.3	MPS II	73.1	61.6	70.5	2441.7	501.0	2441.7	17.0
13.2	MPS II	120.9	84.1	163.4	761.3	192.2	761.3	20.2
13.1	MPS II	54.8	70.8	48.0	1135.3	306.8	1135.3	21.3
15.2	MPS II	49.6	67.4	35.1	787.4	183.3	787.4	18.9
15.5	MPS II	38.7	12.3	24.2	977.1	278.6	977.1	22.2
19.5	MPS II	61.1	62.5	51.8	739.5	204.9	739.5	21.7
18.8	MPS II	46.8	64.5	35.4	409.3	103.9	409.3	20.2
15	MPS II	65.3	70.5	50.9	1336.1	335.3	1336.1	20.1
19	MPS II	138.5	20.8	55.8	1143.5	298.0	1143.5	20.7
15	MPS II	413.2	289.9	594.2	380.0	139.7	380.0	26.9
21.7	MPS II	177.0	100.5	210.3	1317.1	316.2	1317.1	19.4
25	MPS II	36.5	13.0	30.0	496.0	163.1	496.0	24.7
20.5	MPS II	27.0	63.6	25.6	547.9	144.5	547.9	20.9
35	MPS II	27.7	58.6	25.2	677.5	218.5	677.5	24.4
26	MPS II	31.9	58.9	27.3	759.0	153.9	759.0	16.9
23	MPS II	27.7	69.3	22.5	444.2	115.2	444.2	20.6
29	MPS II	165.2	41.1	6.5	971.1	268.5	971.1	21.7
6	MPS IIIA	93.3	30.5	16.1	401.4	101.5	401.4	20.2
9	MPS IIIA	190.3	52.2	32.1	787.9	215.8	787.9	21.5
13	MPS IIIA	94.3	27.6	23.0	401.4	135.1	401.4	25.2
16	MPS IIIA	117.0	33.9	21.7	243.2	42.2	243.2	14.8

17	MPS IIIA	99.4	26.9	11.3	410.7	129.3	410.7	23.9
27	MPS IIIA	85.1	26.5	13.6	331.0	93.6	331.0	22.1
4.5	MPS IIIB	327.2	106.3	36.3	849.5	220.6	849.5	20.6
4	MPS IIIB	189.2	53.0	24.2	701.7	162.4	701.7	18.8
6	MPS IIIB	131.2	46.1	19.2	427.1	116.1	427.1	21.4
8	MPS IIIB	82.8	23.1	15.6	330.6	96.3	330.6	22.6
9	MPS IIIB	116.1	38.4	17.3	321.8	87.0	321.8	21.3
9	MPS IIIB	214.4	74.8	33.8	730.2	207.0	730.2	22.1
7	MPS IIIB	163.1	45.1	27.0	418.3	114.8	418.3	21.5
14.5	MPS IIIB	56.3	14.9	17.9	421.2	86.2	421.2	17.0
12.8	MPS IIIB	212.6	65.1	21.8	929.1	246.3	929.1	21.0
27	MPS IIIB	140.2	44.3	15.3	171.2	57.0	171.2	25.0
24	MPS IIIB	229.7	50.7	27.3	401.1	64.4	401.1	13.8
3.4	MPS IVA	38.2	9.2	2.4	1142.1	322.8	1142.1	22.0
3.6	MPS IVA	15.9	1.3	2.6	642.8	160.2	642.8	20.0
3.380821918	MPS IVA	65.2	13.6	1.1	761.0	272.0	761.0	26.3
6	MPS IVA	102.1	52.9	10.6	319.3	107.8	319.3	25.2
7	MPS IVA	76.6	47.2	8.3	235.1	4.5	235.1	1.9
8	MPS IVA	449.1	226.4	25.2	1278.6	471.4	1278.6	26.9
9	MPS IVA	12.0	20.9	9.8	360.0	106.6	360.0	22.8
6	MPS IVA	35.3	2.7	2.9	894.3	293.9	894.3	24.7
9.3	MPS IVA	11.0	3.3	3.2	412.1	104.4	412.1	20.2
7.75	MPS IVA	45.5	4.3	1.3	997.0	281.2	997.0	22.0
7.739726027	MPS IVA	34.1	8.1	2.5	985.3	287.2	985.3	22.6
6.857534247	MPS IVA	47.3	7.3	3.4	1060.8	314.3	1060.8	22.9
8.8	MPS IVA	43.5	6.4	11.3	1190.5	447.2	1190.5	27.3
6.25205479	MPS IVA	57.1	4.7	1.5	1860.4	595.4	1860.4	24.2
8.4	MPS IVA	23.7	5.2	2.2	814.4	233.7	814.4	22.3

8.4	MPS IVA	22.7	4.8	2.9	1314.7	241.2	1314.7	15.5
8.9	MPS IVA	17.5	3.9	2.3	1159.9	220.5	1159.9	16.0
9.2	MPS IVA	17.7	3.6	2.3	1085.4	215.8	1085.4	16.6
10.5	MPS IVA	18.0	2.6	2.5	858.7	204.6	858.7	19.2
13.4	MPS IVA	12.8	1.1	2.2	422.1	82.8	422.1	16.4
10	MPS IVA	44.7	6.2	3.3	973.5	255.5	973.5	20.8
10	MPS IVA	78.4	36.9	15.5	339.8	183.2	339.8	35.0
12	MPS IVA	130.1	47.3	5.8	270.2	108.8	270.2	28.7
12.3945205	MPS IVA	20.2	3.3	1.4	645.9	143.1	645.9	18.1
12.16438356	MPS IVA	32.0	5.5	1.9	1181.2	313.4	1181.2	21.0
14	MPS IVA	20.7	1.4	1.1	365.9	98.0	365.9	21.1
17.9	MPS IVA	33.1	7.9	2.5	1015.1	230.2	1015.1	18.5
15	MPS IVA	30.9	8.8	21.2	929.2	259.2	929.2	21.8
17	MPS IVA	30.3	8.9	19.3	751.4	174.2	751.4	18.8
16	MPS IVA	32.1	6.0	2.8	1161.4	232.6	1161.4	16.7
16	MPS IVA	23.9	3.1	4.7	500.5	138.7	500.5	21.7
16	MPS IVA	24.0	2.1	6.6	305.2	74.9	305.2	19.7
15.3506849	MPS IVA	30.4	6.9	0.6	937.9	223.9	937.9	19.3
15	MPS IVA	128.6	63.4	9.0	189.3	67.2	189.3	26.2
15	MPS IVA	17.4	6.8	5.2	915.6	201.3	915.6	18.0
17	MPS IVA	16.7	7.5	2.3	644.4	147.1	644.4	18.6
18.09315068	MPS IVA	21.8	3.8	2.5	885.1	139.1	885.1	13.6
28.31506849	MPS IVA	17.2	3.8	2.0	451.3	88.1	451.3	16.3
48	MPS IVA	39.4	12.6	4.4	544.3	238.8	544.3	30.5
56	MPS IVA	16.1	5.4	2.6	265.6	44.2	265.6	14.3
26	MPS IVA	140.8	69.3	17.1	141.0	38.2	141.0	21.3
35	MPS IVA	106.7	52.8	15.2	144.5	63.7	144.5	30.6
12.7	MPS IVB	30.0	7.3	2.1	841.5	165.2	841.5	16.4

12.9	MPS IVB	22.4	4.3	1.9	966.2	229.4	966.2	19.2
17.4	MPS IVB	15.7	7.0	2.9	280.0	65.3	280.0	18.9
17.7	MPS IVB	13.4	3.7	3.7	464.5	98.2	464.5	17.5
18.3	MPS IVB	17.7	2.9	1.7	416.5	66.1	416.5	13.7
30	MPS VII	440.4	157.3	62.5	562.1	137.3	562.1	19.6
29	MPS VII	36.7	7.9	13.0	402.2	148.5	402.2	27.0

Abbreviations: DiHS-0S= *O*-sulfated heparan sulfate, DiHS-NS= *N*-sulfated heparan sulfate, Di-4S= dermatan sulfate, KS= total keratan sulfate, di KS= di-sulfated keratan sulfate, mono KS= mono-sulfated keratan sulfate.

Table S3: Control values for *O*-sulfated heparan sulfate (DiHS-0S) shown in ng/mL.

Age	DiHS-0S
0	107.4
0.0027	49.4
0.0109	104.0
0.0219	129.7
0.0356	113.8
0.0739	122.0
0.08219	23.6
0.08219	117.5
0.08219	111.1
0.083333333	101.8
0.083333333	121.2
0.117808219	63.0
0.12	46.8
0.175342466	114.5
0.246575342	68.2
0.2466	45.3
0.2466	104.4
0.25	120.3
0.320547945	114.7
0.3288	89.4
0.3288	137.2
0.345205479	96.6
0.353424658	66.1
0.36	110.5
0.40	96.9
0.4137	57.8
0.4137	96.6
0.4137	114.4
0.416666667	127.2
0.416666667	121.7
0.42	42.4
0.48	44.0
0.4959	117.3
0.4959	100.4
0.50	81.8
0.56	46.6
0.5808	99.8
0.583333333	88.5
0.666666667	101.2
0.68	80.7
0.70	40.8
0.726027397	82.4
0.7479	50.7
0.7479	123.9
0.7479	123.6
0.7479	79.4
0.75	71.6
0.761643836	73.6
0.8329	63.8
0.8329	62.0
0.87	49.5
0.88	84.2
0.882191781	86.0
0.906849315	60.6
0.9151	65.3
0.916666667	75.5
0.916666667	49.3
1	58.1
1	62.6
1	75.4
1	102.2
1	61.2
1	80.2
1	62.7
1	93.9
1	57.1
1	47.0
1	116.1
1	58.9
1	83.4
1	90.9
1	56.1
1	84.1
1	84.3
1	99.9
1	64.2
1	63.1
1	72.3
1	66.6
1	68.0
1	67.1
1	84.3
1	72.1
1	58.9
1.01	63.2
1.019178082	104.1
1.030136986	61.8
1.03	27.7
1.04	31.4
1.08	84.5
1.101369863	43.9
1.197260274	84.5
1.32	28.4
1.51	23.6
1.539726027	79.7
1.684931507	66.0
1.704109589	82.6
1.74	57.4
1.775342466	72.7
1.83	32.8
1.860273973	89.9
2	48.5
2	101.4
2	92.6
2	42.9
2	71.9
2	98.4
2	64.5
2	53.3
2	89.2

2	85.0
2	79.8
2	77.8
2	60.9
2	72.4
2	72.0
2.035616438	68.3
2.043835616	65.8
2.449315068	96.0
2.60	21.2
2.663013699	110.8
2.67	36.1
2.690410959	40.1
2.690410959	58.3
2.72	36.8
3.0	39.9
3	64.9
3	58.8
3	57.9
3	3.1
3	64.1
3	64.5
3	63.8
3	40.9
3	55.9
3	54.5
3	88.3
3	54.9

3	84.0
3	69.2
3	58.9
3.07	99.0
3.13	24.5
3.309589041	63.0
3.33	29.9
3.676712329	67.7
3.82739726	93.3
3.89	69.2
4	92.0
4	60.0
4	64.7
4	57.9
4	89.4
4	73.5
4	52.2
4.02	28.0
4.25	28.1
4.309589041	62.9
5	82.3
5	55.6
5	50.2
5	84.6
5	63.1
5	94.3
5	48.0
5	85.7

5.265753425	64.5
5.55	33.8
5.99	27.0
6	71.3
6	72.1
6	69.9
6	62.7
6.16	33.0
6.40	27.9
6.805479452	50.4
7	88.9
7	65.2
7	31.6
7	55.4
7	80.7
7	84.9
7	76.2
7	52.0
7	64.3
7	71.5
7.101369863	41.7
7.70	26.6
8	52.3
8	86.5
8	68.6
8	62.8
8	65.8
8	54.3

8	66.4
8	64.7
8	56.7
8.024657534	60.1
8.03	74.3
8.10	19.7
8.11	97.3
8.523287671	59.5
9	42.0
9	96.8
9	68.2
9	75.5
9	60.9
9	49.0
9	66.0
9.512328767	55.6
10	68.5
10.96164384	76.6
11	7.9
11	69.8
11	67.5
11	79.8
11.44931507	73.8
11.96164384	42.2
12	83.9
12	84.4
12	61.7
13	51.7

13	40.4
13	57.2
13	59.1
13	67.2
13.05205479	44.5
14	97.3
14	68.7
14	56.1
14	75.0
14	62.4

14	84.3
14	41.1
14	42.4
14	38.6
14.21	74.8
15	35.9
15	77.9
15	84.4
15	35.7
15	36.3

16	73.1
17	34.6
17	46.2
20	79.8
20	75.3
26	30.9
26	48.6
34	42.9
39	49.3
40	39.7

41	45.5
43	43.5
52	34.3
54	44.5
57	67.3
59	59.5
60	88.0
62	38.1

Table S4: Control values for *N*-sulfated heparan sulfate (DiHS-NS) shown in ng/mL.

Age	DiHS-0S
0	27.5
0	25.2
0	21.6
0.0027	14.5
0.0109	18.2
0.0219	21.0
0.0356	16.7
0.038356164	22.6
0.0384	14.8
0.0739	17.9
0.082	23.5
0.08219	2.0
0.08219	16.1
0.08219	12.1
0.083333333	25.3
0.083333333	1.7
0.117808219	1.5
0.12	0.8
0.166666667	17.4
0.175342466	19.7
0.246575342	10.8
0.2466	27.7
0.2466	7.1
0.2466	12.5
0.25	24.0
0.320547945	28.5
0.3288	9.6
0.3288	14.7
0.345205479	17.4
0.353424658	20.1
0.364383562	20.5
0.36	6.4
0.40	8.2
0.4137	0.8
0.4137	19.8
0.4137	9.8
0.4137	12.4
0.4137	12.4
0.416666667	24.8
0.416666667	16.2
0.416666667	18.2
0.416666667	16.2
0.48	5.7
0.4959	20.9
0.4959	13.7
0.5	24.1
0.50	11.8
0.550684932	19.7
0.5808	11.0
0.5808	13.9
0.583333333	17.2
0.666666667	16.3
0.68	12.5
0.70	5.6
0.726027397	13.1
0.7479	10.1
0.7479	20.7
0.7479	19.2
0.7479	10.7
0.75	7.3
0.761643836	10.8
0.8329	1.5
0.8329	9.0
0.8329	8.1
0.88	3.8
0.882191781	11.9
0.906849315	9.9
0.9151	20.1
0.9151	8.4
0.916666667	11.8
0.916666667	6.5
1	9.7
1	7.9
1	2.9
1	16.6
1	12.7
1	13.3
1	10.6
1	22.6
1	4.4
1	1.2
1	12.0
1	5.3
1	16.7
1	2.4
1	3.9
1	10.0
1	11.9
1	17.9
1	11.3
1	11.2
1	3.3
1	8.3
1	11.0
1	9.2
1	8.8
1	8.2
1	9.3
1	4.8
1	5.3
1.01	9.9
1.019178082	15.9
1.030136986	13.9
1.03	7.7
1.04	4.6
1.08	9.7
1.101369863	1.6

1.197260274	1.0
1.32	6.1
1.51	4.7
1.539726027	14.0
1.684931507	21.6
1.704109589	12.2
1.74	7.4
1.775342466	9.4
1.83	5.4
2	8.4
2	18.9
2	14.7
2	5.3
2	0.7
2	18.8
2	1.4
2	9.6
2	12.3
2	10.2
2	9.3
2	8.1
2	5.9
2	7.7
2	10.2
2.035616438	10.5
2.043835616	9.0
2.232876712	16.6
2.449315068	13.0
2.60	0.5
2.663013699	15.7
2.663013699	18.8
2.67	7.9
2.690410959	7.4
2.690410959	15.4
2.72	7.6
3.0	6.4
3	5.4
3	5.6
3	12.5
3	0.5
3	8.4
3	4.7
3	5.5
3	4.4
3	6.9
3	7.2
3	17.7
3	7.7
3	10.1
3	9.0
3	4.0
3.07	5.8
3.13	19.2
3.309589041	12.3
3.33	8.6
3.676712329	14.1
3.82739726	16.3
3.89	3.3
4	10.8
4	8.3
4	16.9
4	5.1
4	8.9
4	7.3
4	10.9
4	7.2
4.02	6.2
4.25	0.7
4.309589041	7.2
5	9.6
5	6.6
5	8.5
5	13.5
5	3.2
5	11.3
5.265753425	11.3
5.55	6.4
5.99	5.1
6	6.9
6	10.4
6	5.5
6	9.8
6.16	7.7
6.40	6.0
6.805479452	11.8
7	12.4
7	1.5
7	9.0
7	8.4
7	9.9
7	8.7
7	6.4
7	9.3
7	4.7
7.101369863	3.9
7.70	6.2
8	7.8
8	10.2
8	15.1
8	7.1
8	5.7
8	6.4
8	13.9
8	5.3
8	9.4
8.024657534	7.0
8.03	11.1
8.10	7.9
8.11	2.9
8.523287671	5.6
9	5.5
9	9.5

9	13.1
9	10.3
9	8.4
9	6.7
9	8.5
9.512328767	7.9
10	12.1
10	7.0
10.96164384	9.0
11	7.5
11	8.6
11	10.9
11	8.2
11	5.3

11.44931507	10.2
11.96164384	6.5
12	13.5
12	10.1
13	9.7
13	3.7
13	6.0
13	6.7
13	5.7
13.05205479	5.7
14	9.2
14	12.0
14	7.9
14	9.0

14	13.4
14	6.4
14	5.0
14	9.1
14.21	7.7
15	7.1
15	14.7
15	5.5
15	3.8
16	15.1
17	6.7
17	5.6
20	11.6
20	7.3

26	4.6
26	5.6
34	4.6
39	4.6
40	6.2
41	7.5
43	9.8
52	6.4
54	5.7
57	11.0
59	9.8
60	12.0
62	4.8

Table S5: Control values for dermatan sulfate (Di-4S) shown in ng/mL.

Age	Di-4S
0	18.6
0	27.2
0	25.7
0.0027	12.4
0.0109	9.0
0.0109	36.5
0.0219	34.4
0.0739	26.9
0.082	28.5
0.08219	16.8
0.08219	18.9
0.08219	20.2
0.083333333	25.2
0.083333333	27.3
0.117808219	22.3
0.12	30.3
0.166666667	23.6
0.166666667	38.1
0.175342466	31.3
0.246575342	25.2
0.2466	25.2
0.2466	4.6
0.2466	4.2
0.25	19.6
0.320547945	29.2
0.3288	28.3
0.3288	15.4
0.3288	29.6
0.333333333	30.0
0.345205479	19.0
0.353424658	24.2
0.364383562	31.3
0.36	21.5
0.40	16.6
0.4137	1.6
0.4137	30.6
0.4137	30.3
0.4137	20.7
0.4137	26.5
0.4137	21.7
0.416666667	30.9
0.416666667	35.2
0.416666667	13.4
0.416666667	32.3
0.42	20.7
0.48	26.5
0.4959	9.5
0.4959	16.7
0.5	19.3
0.50	23.4
0.550684932	19.4
0.56	21.8
0.5808	17.5
0.5808	30.2
0.583333333	11.6
0.666666667	24.7
0.68	11.6
0.70	15.1
0.726027397	27.4
0.7479	12.5
0.7479	27.8
0.7479	23.6
0.7479	21.5
0.75	19.4
0.761643836	19.4
0.8329	39.3
0.8329	18.5
0.8329	17.0
0.87	35.7
0.88	27.9
0.882191781	22.9
0.906849315	13.1
0.9151	12.1
0.916666667	21.0
0.916666667	6.8
1	6.0
1	19.7
1	14.1
1	23.3
1	22.5
1	19.8
1	25.3
1	23.0
1	27.9
1	30.6
1	24.0
1	30.8
1	5.4
1	32.8
1	20.4
1	17.6
1	28.5
1	25.2
1	26.3
1	22.8
1	33.4
1	22.0
1	18.5
1	19.9
1	20.4
1	40.1
1	16.3
1	18.7

1	19.2
1	22.9
1	5.4
1.01	19.1
1.019178082	14.1
1.030136986	21.8
1.03	21.6
1.04	8.9
1.08	16.3
1.101369863	21.3
1.197260274	24.0
1.32	20.5
1.51	22.7
1.539726027	20.6
1.684931507	23.3
1.704109589	20.8
1.74	12.2
1.775342466	21.6
1.83	19.0
1.860273973	9.1
2	8.3
2	15.6
2	15.1
2	25.4
2	21.0
2	26.2
2	18.6
2	13.8

2	35.5
2	23.4
2	3.4
2	17.8
2	5.8
2	23.1
2	11.2
2.035616438	20.4
2.043835616	28.0
2.232876712	34.3
2.449315068	11.1
2.60	17.6
2.663013699	15.5
2.663013699	33.6
2.67	17.5
2.690410959	12.9
2.690410959	24.0
2.72	12.3
3.0	16.2
3	32.9
3	7.8
3	1.5
3	13.0
3	0.8
3	15.4
3	1.1
3	29.2
3	25.0

3	14.6
3	14.0
3	24.1
3	17.9
3	16.9
3	27.0
3	24.6
3	18.1
3.07	30.7
3.13	13.5
3.309589041	18.3
3.33	32.9
3.676712329	15.9
3.82739726	29.5
3.89	23.0
4	23.9
4	20.4
4	19.6
4	25.1
4	20.6
4	33.0
4	27.9
4.02	18.7
4.25	20.1
4.309589041	20.2
5	29.3
5	2.7
5	14.9

5	29.3
5	16.9
5	27.5
5	23.5
5	29.4
5	13.1
5	19.1
5.265753425	19.9
5.55	24.3
5.99	22.3
6	24.8
6	24.1
6	22.8
6	18.5
6	19.8
6	19.7
6.16	22.2
6.40	22.2
6.805479452	12.7
7	12.0
7	19.6
7	2.2
7	11.6
7	17.8
7	19.7
7	19.9
7	16.2
7	19.6

7	11.1
7.101369863	14.3
7.70	31.1
8	22.0
8	26.1
8	29.2
8	16.5
8	16.3
8	29.9
8	24.3
8	25.7
8	18.0
8	4.6
8.024657534	21.7
8.03	20.1
8.10	15.3
8.11	29.9

8.523287671	33.1
9	16.1
9	31.3
9	22.1
9	16.9
9	17.0
9	15.8
9	14.0
9.512328767	21.0
10	20.6
10	19.2
10	25.0
10.96164384	22.9
11	1.4
11	21.2
11	27.6
11	20.8

11	16.6
11	24.8
11.44931507	21.6
11.96164384	12.6
12	2.2
12	18.5
13	19.1
13	22.0
13	20.9
13	18.8
13	17.2
13.05205479	20.1
14	4.2
14	18.9
14	20.8
14	18.2
14	21.2

14	16.7
14.21	21.4
15	1.1
15	15.5
15	20.8
15	9.7
15	16.9
17	24.8
17	19.6
20	44.1
20	14.7
26	20.1
26	15.6
34	11.6
39	70.1
40	14.5
43	3.8

Table S6: Mono-sulfated keratan sulfate (KS) control values shown in ng/mL.

Age	Mono-sulfated KS
0.0	643.3
0.0	553.1
0.0	549.5
0.0	384.9
0.0	497.9
0.0	912.1
0.0	760.5
0.0	1095.1
0.0	550.7
0.1	1015.0
0.1	1171.5
0.1	252.8
0.1	654.8
0.1	783.3
0.1	955.7
0.1	838.4
0.1	343.1
0.1	513.2
0.2	699.4
0.2	1071.1
0.2	1002.1
0.2	584.6
0.2	818.9
0.2	554.3
0.2	1193.0
0.2	945.8
0.2	1166.4
0.2	637.3
0.2	499.3
0.3	918.1
0.3	608.0
0.3	967.2
0.3	979.0
0.3	1075.8
0.3	519.4
0.3	524.2
0.4	570.5
0.4	1109.8
0.4	421.1
0.4	420.3
0.4	266.4
0.4	769.4
0.4	909.6
0.4	687.4
0.4	741.9
0.4	789.3
0.4	662.9
0.4	1165.2
0.4	762.0
0.4	674.3
0.4	725.2
0.5	544.0
0.5	1005.0
0.5	592.0
0.5	454.8
0.5	488.4
0.5	846.1
0.5	550.5
0.6	940.7
0.6	672.6
0.6	750.9
0.6	603.9
0.6	675.8
0.7	874.6
0.7	557.9
0.7	782.6
0.7	461.2
0.7	334.2
0.7	452.8
0.7	867.8
0.7	483.6
0.7	841.1
0.7	572.7
0.7	607.8
0.8	508.8
0.8	941.5
0.8	325.1
0.8	931.4
0.8	769.6
0.9	607.1
0.9	729.2
0.9	396.7
0.9	717.1
0.9	818.0
0.9	551.6
0.9	942.1
0.9	630.8
0.9	324.0
1.0	319.1
1.0	476.4
1.0	871.1
1.0	1190.9
1.0	830.3
1.0	798.1
1.0	1071.7
1.0	1095.1
1.0	605.9
1.0	663.1
1.0	389.7
1.0	451.9
1.0	1031.7
1.0	854.8
1.0	749.8
1.0	1150.2
1.0	1061.3
1.0	714.2

1.0	489.4
1.0	724.8
1.0	825.2
1.0	1124.3
1.0	992.4
1.0	632.0
1.0	840.2
1.0	988.8
1.0	934.9
1.0	954.4
1.0	828.3
1.0	581.3
1.0	576.9
1.0	732.8
1.0	784.4
1.0	786.2
1.0	749.8
1.01	697.4
1.02	495.0
1.03	393.6
1.03	386.1
1.04	967.9
1.08	558.9
1.10	597.0
1.20	723.3
1.30	768.0
1.32	407.7
1.51	634.5

1.54	878.5
1.68	617.5
1.70	857.6
1.74	515.9
1.78	892.4
1.83	845.6
2.00	929.7
2.00	917.4
2.00	659.1
2.00	509.1
2.00	861.9
2.00	768.2
2.00	448.7
2.00	1111.5
2.00	821.5
2.00	1153.1
2.00	579.6
2.00	670.4
2.00	746.9
2.00	871.2
2.00	1044.2
2.04	1060.2
2.23	1156.4
2.45	424.3
2.56	850.3
2.60	612.3
2.66	876.1
2.66	736.3

2.67	719.1
2.69	375.4
2.69	917.2
2.98	1198.7
3.00	419.8
3.00	515.8
3.00	626.5
3.00	660.4
3.00	659.4
3.00	853.1
3.00	364.2
3.00	764.7
3.00	689.4
3.00	435.2
3.00	641.8
3.00	763.3
3.00	757.2
3.00	575.7
3.00	293.5
3.00	811.6
3.00	896.9
3.00	932.5
3.00	901.6
3.00	787.9
3.13	604.0
3.31	817.8
3.33	612.3
3.68	1042.3

3.83	754.1
3.89	687.9
4.00	1032.6
4.00	697.1
4.00	622.4
4.00	582.7
4.00	399.5
4.00	1054.5
4.00	611.1
4.02	775.3
4.25	546.9
4.31	660.0
5.00	923.8
5.00	676.8
5.00	1067.8
5.00	686.4
5.00	586.0
5.00	635.4
5.00	656.8
5.00	764.2
5.00	1025.4
5.3	659.6
5.6	324.6
6.0	907.8
6.0	639.1
6.0	643.4
6.0	710.5
6.0	719.1

6.0	705.4
6.0	910.3
6.2	518.8
6.4	652.8
6.8	757.7
7.0	456.1
7.0	973.1
7.0	527.8
7.0	769.7
7.0	925.8
7.0	981.5
7.0	833.7
7.0	711.8
7.0	966.6
7.0	591.5
7.1	666.0
7.7	589.4
8.0	905.0
8.0	648.5
8.0	283.6
8.0	859.7
8.0	752.9
8.0	590.0
8.0	422.8

8.0	857.1
8.0	832.6
8.0	744.3
8.0	676.3
8.0	1008.7
8.0	840.7
8.1	669.4
8.1	607.4
8.5	696.5
9.0	728.7
9.0	491.6
9.0	354.1
9.0	452.2
9.0	1043.9
9.0	692.7
9.0	712.7
9.0	670.9
9.5	484.7
10.0	663.6
11.0	484.7
11.0	561.3
11.0	359.6
11.0	840.2
11.0	470.1

11.4	722.6
12.0	807.6
12.0	504.1
12.0	340.8
12.0	217.8
12.0	832.1
12.0	457.7
13.0	806.5
13.0	437.6
13.0	562.0
13.0	594.9
13.0	722.0
13.0	777.4
13.1	687.7
14.0	422.8
14.0	350.5
14.0	655.7
14.0	640.8
14.0	296.4
14.0	449.8
14.0	376.8
14.0	388.2
14.0	525.3
14.0	589.0

14.0	392.3
14.2	526.3
15.0	307.5
15.0	296.7
15.0	329.2
15.0	374.3
15.0	316.4
16.0	469.0
17.0	148.6
17.0	396.5
20.0	266.4
26	196.1
26	204.6
34	424.1
39	368.6
40	226.5
41	204.5
43	369.7
52	182.8
54	334.3
59	286.9
60	280.7
62	305.2

Table S7: Control values for di-sulfated keratan sulfate shown in ng/mL.

Age	Di-sulfated KS
0.0	106.3
0.0	82.0
0.0	59.2
0.0	61.6
0.0	235.4
0.0	147.7
0.0	94.5
0.0	260.6
0.0	89.2
0.1	281.2
0.1	132.3
0.1	117.0
0.1	86.4
0.1	117.4
0.1	162.8
0.1	97.7
0.1	80.6
0.1	80.4
0.2	232.7
0.2	166.0
0.2	92.5
0.2	240.9
0.2	74.4
0.2	129.1
0.2	86.9
0.3	153.7
0.3	122.8
0.3	100.2
0.3	157.6
0.3	187.9
0.3	94.4
0.4	78.8
0.4	194.1
0.4	22.2
0.4	108.4
0.4	15.1
0.4	216.3
0.4	148.7
0.4	85.8
0.4	81.1
0.4	117.5
0.4	140.9
0.4	126.0
0.5	87.3
0.5	235.7
0.5	107.6
0.5	77.9
0.5	171.8
0.5	199.3
0.6	161.4
0.6	107.7
0.6	242.4
0.6	152.7
0.6	110.7
0.7	239.3
0.7	175.1
0.7	98.3
0.7	108.7
0.7	48.7
0.7	144.8
0.7	94.7
0.7	192.9
0.7	111.1
0.7	111.1
0.8	144.7
0.8	259.7
0.8	137.0
0.8	118.5
0.8	138.2
0.9	93.9
0.9	178.4
0.9	63.1
0.9	157.7
0.9	88.4
0.9	124.3
0.9	73.4
1.0	54.7
1.0	172.6
1.0	138.4
1.0	189.7
1.0	125.5
1.0	120.5
1.0	121.8
1.0	165.7
1.0	94.0
1.0	191.6
1.0	212.5
1.0	180.2
1.0	260.3
1.0	208.7
1.0	166.0
1.0	54.6
1.0	121.1
1.0	100.6
1.0	83.8
1.0	139.0
1.0	102.0
1.0	124.3
1.0	115.4
1.0	210.4
1.0	100.7
1.0	100.6
1.0	116.8
1.0	132.8
1.0	208.7
1.0	169.8
1.0	85.3

1.0	62.0
1.0	84.9
1.1	199.7
1.1	112.4
1.2	129.8
1.3	285.1
1.3	68.7
1.5	102.8
1.5	134.0
1.7	100.9
1.7	139.0
1.7	198.6
1.8	133.3
1.8	151.6
1.9	183.1
2.0	162.7
2.0	174.3
2.0	257.3
2.0	73.7
2.0	172.0
2.0	225.9
2.0	120.0
2.0	124.7
2.0	81.8
2.0	156.4
2.0	130.4
2.0	143.8

2.0	128.0
2.0	133.6
2.0	178.1
2.2	174.9
2.4	149.8
2.6	263.2
2.6	113.1
2.7	229.8
2.7	134.6
2.7	87.5
2.7	201.5
2.7	195.3
3.0	184.2
3.0	88.5
3.0	104.6
3.0	104.0
3.0	118.0
3.0	75.5
3.0	285.0
3.0	175.3
3.0	199.9
3.0	288.0
3.0	245.0
3.0	133.3
3.0	137.1
3.0	134.4
3.0	121.4

3.1	220.9
3.1	126.2
3.3	123.8
3.3	118.6
3.7	188.1
3.8	237.5
3.9	238.7
4.0	210.6
4.0	119.2
4.0	278.5
4.0	148.8
4.0	99.3
4.0	160.7
4.0	138.6
4.0	149.4
4.3	120.9
4.3	274.3
5.0	194.9
5.0	111.7
5.0	253.0
5.0	241.9
5.0	184.9
5.0	111.0
5.0	176.8
5.3	105.4
5.6	61.9
6.0	213.3

6.0	226.6
6.0	330.2
6.0	160.7
6.0	140.7
6.0	304.7
6.2	112.8
6.4	125.1
6.8	171.9
7.0	115.9
7.0	213.4
7.0	73.1
7.0	160.5
7.0	166.8
7.0	116.1
7.0	139.6
7.1	157.0
7.7	128.4
8.0	162.7
8.0	250.6
8.0	147.1
8.0	159.5
8.0	162.5
8.0	148.1
8.0	153.1
8.0	132.5
8.0	146.2
8.0	152.4

8.0	175.6
8.0	292.6
8.1	121.4
8.1	271.9
8.5	160.2
9.0	162.6
9.0	154.9
9.0	201.2
9.0	161.4
9.0	147.8
9.0	175.2
9.0	110.6
9.5	214.6
10.0	243.5
11.0	4.8
11.0	273.6
11.0	194.2

11.0	116.0
11.0	95.4
12.0	145.1
12.0	101.5
12.0	228.0
12.0	152.4
12.0	204.2
12.0	192.6
13.0	190.7
13.0	278.4
13.0	69.6
13.0	92.3
13.0	122.3
13.0	266.1
13.1	124.8
14.0	232.4
14.0	101.2

14.0	146.7
14.0	122.7
14.0	96.4
14.0	282.9
14.0	90.8
14.0	115.1
14.0	78.4
14.0	100.9
14.0	67.8
14.2	181.8
15.0	81.0
15.0	74.5
15.0	255.6
15.0	69.0
15.0	51.7
16.0	118.9
17.0	70.5

17.0	71.4
20.0	131.4
20.0	206.7
26	43.3
26	32.6
34	86.8
39	53.8
40	33.4
41	64.9
43	111.7
52	77.1
54	86.8
57	93.3
59	106.9
60	62.5
62	102.2

Table S8: Control values for the ratio of di-sulfated keratan sulfate (KS) to total keratan sulfate.

Age	Di-sulfated KS/Total KS
0.0	14.18
0.0	12.91
0.0	9.72
0.0	13.81
0.0	13.94
0.0	11.06
0.0	19.22
0.0	13.93
0.1	21.70
0.1	10.15
0.1	11.66
0.1	13.03
0.1	14.55
0.1	10.43
0.1	19.03
0.1	13.54
0.2	24.97
0.2	13.42
0.2	13.66
0.2	22.73
0.2	5.99
0.2	16.85
0.2	14.82
0.3	14.34
0.3	16.80
0.3	9.28
0.3	12.78
0.3	26.57
0.3	15.26
0.4	12.14
0.4	14.88
0.4	5.01
0.4	20.51
0.4	5.35
0.4	21.94
0.4	17.78
0.4	9.80
0.4	10.90
0.4	13.36
0.4	17.29
0.4	14.80
0.5	13.83
0.5	19.14
0.5	13.75
0.5	16.88
0.5	26.58
0.6	14.64
0.6	13.80
0.6	24.40
0.6	20.18
0.6	14.08
0.7	21.49
0.7	23.89
0.7	11.16
0.7	19.08
0.7	12.73
0.7	24.24
0.7	16.37
0.7	18.65
0.7	16.24
0.7	15.45
0.8	22.14
0.8	21.62
0.8	11.29
0.8	15.22
0.9	13.40
0.9	19.66
0.9	13.73
0.9	18.03
0.9	13.81
0.9	11.66
0.9	18.47
1.0	14.63
1.0	26.60
1.0	13.71
1.0	13.74
1.0	13.13
1.0	13.11
1.0	10.20
1.0	13.14
1.0	13.43
1.0	22.41
1.0	23.34
1.0	21.77
1.0	18.86
1.0	10.04
1.0	14.32
1.0	9.20
1.0	11.70
1.0	14.20
1.0	9.84
1.0	11.53
1.0	12.23
1.0	26.57
1.0	14.86
1.0	12.07
1.0	12.97
1.0	14.45
1.0	21.77
1.01	19.58
1.03	17.81
1.03	13.84
1.04	8.06
1.08	26.33
1.10	15.84
1.20	15.22

1.30	27.07
1.32	14.42
1.51	13.94
1.54	13.24
1.68	14.05
1.70	13.95
1.78	12.99
1.83	15.20
1.86	12.96
2.00	14.90
2.00	12.67
2.00	21.90
2.00	10.06
2.00	25.26
2.00	20.77
2.00	13.51
2.00	21.75
2.00	9.06
2.00	11.95
2.00	18.37
2.00	17.66
2.00	14.63
2.00	13.30
2.00	14.57
2.23	13.14
2.45	26.09
2.56	23.64
2.60	15.59

2.66	23.78
2.67	15.77
2.69	18.90
2.69	18.01
2.72	13.70
3.0	13.32
3.00	17.42
3.00	16.86
3.00	14.24
3.00	15.16
3.00	10.27
3.00	25.04
3.00	20.72
3.00	29.47
3.00	12.94
3.00	12.82
3.00	12.97
3.00	13.36
3.07	16.12
3.13	17.28
3.31	13.14
3.33	16.23
3.68	15.29
3.83	23.95
3.89	25.76
4.00	16.94
4.00	14.60
4.00	20.34

4.00	19.91
4.00	13.22
4.00	18.49
4.02	16.16
4.25	18.10
4.31	29.36
5.00	17.42
5.00	14.16
5.00	26.93
5.00	29.22
5.00	22.54
5.00	12.69
5.00	14.71
5.3	13.78
5.6	16.02
6.0	19.02
6.0	26.17
6.0	18.44
6.0	16.37
6.0	25.08
6.2	17.86
6.4	16.08
6.8	18.49
7.0	20.26
7.0	17.99
7.0	12.16
7.0	14.05
7.0	16.67

7.0	14.02
7.0	19.09
7.1	19.08
7.7	17.88
8.0	15.23
8.0	27.87
8.0	17.48
8.0	21.60
8.0	25.94
8.0	15.16
8.0	13.73
8.0	16.42
8.0	18.39
8.0	14.83
8.0	25.82
8.1	15.35
8.1	30.92
8.5	18.70
9.0	18.25
9.0	23.96
9.0	26.31
9.0	17.58
9.0	19.74
9.0	14.16
9.5	30.68
10.0	26.85
11.0	0.99
11.0	32.77

11.0	35.07
11.0	12.13
11.0	16.87
12.0	15.23
12.0	16.76
12.0	19.71
13.0	19.12
13.0	38.88
13.0	11.02
13.0	13.43
13.0	14.48
13.0	25.50

13.1	15.36
14.0	35.47
14.0	22.40
14.0	18.28
14.0	16.07
14.0	24.54
14.0	38.61
14.0	19.42
14.0	22.88
14.0	12.98
14.0	14.63
14.0	14.73

14.2	25.67
15.0	20.85
15.0	20.07
15.0	15.56
15.0	14.05
16.0	20.22
17.0	32.16
17.0	15.26
20.0	19.37
26	18.10
26	13.74
34	17.00

39	12.74
40	12.84
41	24.09
43	23.19
52	29.67
54	20.62
57	15.39
59	27.15
60	18.20
62	25.10
